# Gene therapy by virus-like self-spooling toroidal DNA condensates for revascularization of hindlimb ischemia

**DOI:** 10.1186/s12951-024-02620-3

**Published:** 2024-07-15

**Authors:** Yue Wang, Jun Liu, Changgui Tong, Lei Li, Hongyang Cui, Liuwei Zhang, Ming Zhang, Shijia Zhang, Kehui Zhou, Xiabin Lan, Qixian Chen, Yan Zhao

**Affiliations:** 1 Department of Gastric Surgery, Cancer Hospital of China Medical University, No. 44 Xiaoheyan Road, Dadong District, Shenyang City, Liaoning 110042 China; 2 Department of Gastric Surgery, Cancer Hospital of Dalian University of Technology, No. 44 Xiaoheyan Road, Dadong District, Shenyang City, Liaoning 110042 China; 3 Provincial Key Laboratory of Interdisciplinary Medical Engineering for Gastrointestinal Carcinoma, Liaoning Cancer Hospital & Institute, No. 44 Xiaoheyan Road, Dadong District, Shenyang City, Liaoning 110042 China; 4 Department of Materials Science and Engineering, Tsinghua University, Beijing City, 100084 China; 5 Innovation Center of Yangtze River Delta, Zhejiang University, Jiaxing, Zhejiang 314100 China; 6 Department of Vascular Surgery, The Second Affiliated Hospital of Dalian Medical University, Dalian, Liaoning 116023 China; 7 Department of Thyroid Surgery, Zhejiang Cancer Hospital, Hangzhou, Zhejiang 310022 China; 8 Hangzhou Institute of Medicine (HIM), Chinese Academy of Sciences, Hangzhou, Zhejiang 310022 China; 9 Key Laboratory of Head & Neck Cancer Translational Research of Zhejiang Province, Hangzhou, Zhejiang 310022 China; 10 Postgraduate Training Base Alliance of Wenzhou Medical University (Zhejiang Cancer Hospital), Hangzhou, Zhejiang 310022 China

**Keywords:** Ischemia, Vascular endothelial growth factors, Angiogenesis, Virus-like gene delivery systems, DNA condensates

## Abstract

Peripheral arterial diseases (PAD) have been reported to be the leading cause for limb amputations, and the current therapeutic strategies including antiplatelet medication or intervene surgery are reported to not clinically benefit the patients with high-grade PAD. To this respect, revascularization based on angiogenetic vascular endothelial growth factor (VEGF) gene therapy was attempted for the potential treatment of critical PAD. Aiming for transcellular delivery of VEGF-encoding plasmid DNA (pDNA), we proposed to elaborate intriguing virus-like DNA condensates, wherein the supercoiled rigid micrometer-scaled plasmid DNA (pDNA) could be regulated in an orderly fashion into well-defined nano-toroids by following a self-spooling process with the aid of cationic block copolymer poly(ethylene glycol)-polylysine at an extraordinary ionic strength (NaCl: 600 mM). Moreover, reversible disulfide crosslinking was proposed between the polylysine segments with the aim of stabilizing these intriguing toroidal condensates. Pertaining to the critical hindlimb ischemia, our proposed toroidal VEGF-encoding pDNA condensates demonstrated high levels of VEGF expression at the dosage sites, which consequently contributed to the neo-vasculature (the particularly abundant formation of micro-vessels in the injected hindlimb), preventing the hindlimb ischemia from causing necrosis at the extremities. Moreover, excellent safety profiles have been demonstrated by our proposed toroidal condensates, as opposed to the apparent immunogenicity of the naked pDNA. Hence, our proposed virus-like DNA condensates herald potentials as gene therapy platform in persistent expressions of the therapeutic proteins, and might consequently be highlighted in the management of a variety of intractable diseases.

**Supplementary Information:**

The online version contains supplementary material available at 10.1186/s12951-024-02620-3.

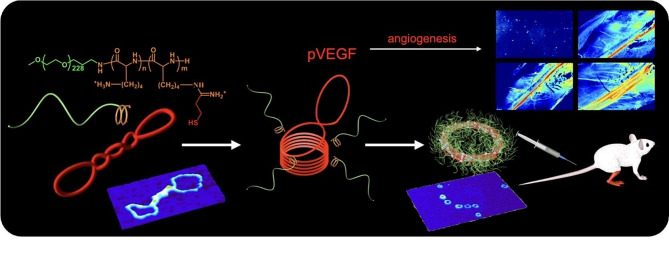

## Introduction

Critical limb ischemia is a severe condition associated with peripheral artery occlusive diseases that manifests as lower limb gangrene, ulceration, and ischemic rest pain. Patients with critical limb ischemia experience decreased perfusion in the distal portion of their lower limbs due to macrovascular lesions. This significantly impairs the target tissue’s capacity to exchange microcirculation and absorb nutrients from the blood. Consequently, peripheral arterial disorders are considered to be one of the most intractable occlusive cardiovascular disease groups and the leading causes for limb amputations [1, 2]. To date, no pharmacological treatment has been verified as capable of preventing peripheral arterial diseases (e.g., limb ischemia) from leading to amputations and mortality [3, 4]. Endovascular intervention to channelize the blocked blood vessels or surgical bypass is the current treatment for patients with critical limb ischemia [5, 6], but these attempts are apparently infeasible when applied to complicated late-stage limb ischemia [7, 8]. Therefore, the development of a novel therapeutic approach to address critical limb ischemia is urgently required.

Vascular endothelial growth factors (VEGFs)-based revascularization therapy is deemed to be a potentially valid approach in the treatment of late-stage limb ischemia [9, 10]; it is envisioned to stimulate the development of neo-vasculature rather than channelize the blocked blood vessels [11]. In principle, VEGFs can be cataloged as a family of the crucial regulators for physiological angiogenesis during embryogenesis, skeletal growth and reproductive activities [4, 12]. For instance, VEGFs have been demonstrated as vital in promoting the proliferation of the cultured vascular endothelial cells derived from arteries, veins and lymphatics [13]. Hence, VEGF-based therapeutics have been attempted in revascularization therapy [6]. Nonetheless, VEGFs when applied to conventional proteinic therapeutics undergo great difficulty in successfully formulating robust neo-vasculature systems, which is conjectured as attributable to the limited retention of the dosed proteinic therapeutics at the pathological site [14]. Hence, it is believed that the persistent local retention of angiogenic VEGF is critical to accomplishing revascularization. To this end, we proposed plasmid DNA (pDNA) as the preferred long-term gene expressing vector for pursuing the persistent expression of VEGF A to accomplish adequate revascularization for treatment of hindlimb ischemia.

Since plasmid DNA (pDNA) is not internalized by the cells due to its large-scale and negative charge, and due to its vulnerability to nuclease degradation [15, 16]. To achieve transcellular delivery of the intact therapeutic pDNA into the host cells, nano-devices as delivery carriers are imperative to elaborate. Indeed, the development of intelligent nano-devices to pursue the breakthrough in medical care—known as nanomedicine—has been spurred by notable advancements in nanotechnology-based biomaterials. Particularly, medical diagnosis and therapy have significantly improved thanks to these medical nano-devices, which are made using versatile chemistry-based engineering to achieve specific functionality [17, 18]. An important instance is gene delivery carrier, which is characterized as nanoparticles that are loaded with genomic material and are intended to be delivered to the diseased region for functional protein expression.

Previously, we have created a poly(ethylene glycol) (PEG)-shielded gene delivery system called polyplex micelles, which is a potentially effective formulation based on the self-assembly of plasmid DNA (pDNA) and PEG-polycation block copolymers [19, 20]. After this electrostatic complexation, pDNA is bundled into a unique rod-shaped bundle. The core compartment of this bundle is made up of pDNA that has been folded multiple times, while the outermost PEG chains act as a protective shell compartment. Recently, we found the condensation pattern of DNA strands are correlated with the expression activities of the condensed DNA.

**Scheme 1:**
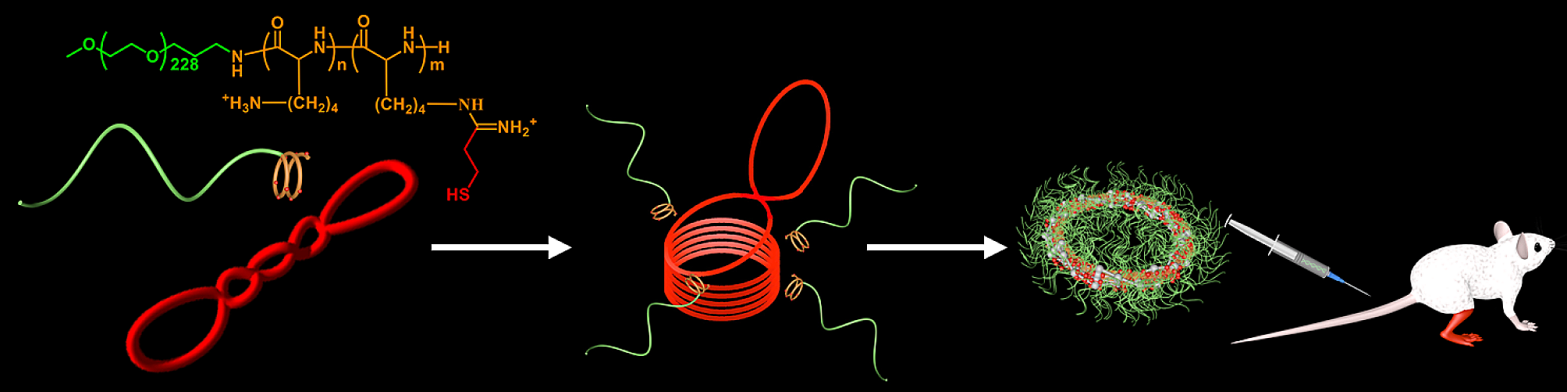
Fabrication of virus-like pDNA condensates for angiogenesis in treatment of hindlimb ischemia, where the supercoiled rigid micrometer-scaled pDNA (VEGF-encoding) macromolecules could be regulated in an orderly fashion into well-defined nano-toroids by following a self-spooling process with the aid of cationic block copolymer poly(ethylene glycol)-polylysine

Of note, DNA condensation is a key phenomenon in genome packaging within nuclei, wherein anionic DNA was regulated by the cationic proteins of histones to induce windings of DNA strands into side-by-side DNA coils. Consequently, the occupied volume of nuclear DNA was decreased by millions of folds. Learning from the natural DNA condensation, we have developed an intriguingly well-defined nanoscaled toroidal VEGF A-encoding plasmid condensate resembling the natural DNA condensing process (Scheme 1). Particularly, the giant plasmid DNA (pDNA) was indicated to undergo spontaneously self-package via looping into an ordered DNA condensate with the aid of a cationic block copolymer of poly(ethylene glycol)-polylysine (PEG-PLys). This DNA condensate possessing intriguing natural DNA packaging scenario is expected to endow favorable biological activities. As a consequence of drastic volume-down transition and charge neutralization from DNA-looping packaging, ready cellular internalization. Furthermore, to pursue practical gene therapy applications, we proposed the formation of disulfide crosslinking between polylysine segments by introducing thiols into the side-chains of lysine units, aiming for stabilizing this intriguing virus-like self-spooling plasmid condensate in the harsh biological environment. Note that the disulfide bond is stable in the extracellular compartment but readily cleavable to form thiols in the glutathione-enriched intracellular microenvironment [20–23]. Consequently, its prolonged survival in the dosed hindlimb through structural crosslinking for the progressive internalization of plasmid DNA is envisioned, while the selective cleavage of the disulfide bonds into the free thiols might facilitate structural disassembly to liberate the plasmid DNA for access by the transcription and translation machinery. Eventually, the consistent expression and secretion of angiogenic VEGFs is speculated from this carefully elaborated virus-like DNA condensates to lead to the formation of neovasculature to relieve hindlimb ischemia.

## Materials and methods

The supplier of α-Methoxy-ω-amino-PEG (10 kDa) was SinoPEG Co. located in Xiamen, China. The supplier of N-carboxyanhydride [Lys(TFA)-NCA] was Hubei Jusheng Co. (Wuhan, China). Wako Pure Chemical Industries, Ltd. was the supplier of dithiothreitol (DTT), diethylenetriamine (DET), N, N-dimethylformamide (DMF), dichloromethane, benzene, and trifluoroacetic acid. Santa Cruz Biotechnology, Inc. (Santa Cruz, CA) provided the N-succinimidyl-3-(2-pyridyldithiol)propionate (SPDP). We obtained the fetal bovine serum (FBS) from Dainippon Sumitomo Parma Co., Ltd. in Osaka, Japan. Promega Co. (Madison, WI) supplied the luciferase assay system kit and cell culture lysis solution. Sigma-Aldrich provided Dulbecco with its modified Eagle’s medium (DMEM) (St. Louis, MO). Regarding the cellular uptake and intracellular distribution experiment, pDNA was labeled with Cy5 in accordance with the manufacturer’s procedure using a Label IT Nucleic Acid Labeling Kit from Mirus Bio Corporation (Madison, WI). A UV detector and a Superdex200 10/300 GL (GE Healthcare, Tokyo, Japan)-equipped LC-2000 system (JASCO, Tokyo, Japan) were utilized for characterizations in the aqueous phase SEC. Therapeutic pDNA of pVEGFA165 (GenBank Accession No. NM 009505, referred to be VEGF hereafter) was used in this study and purchased from Miaoling Biology (Wuhan, China). All animal experimental procedures were performed in accordance with the Guide for the Care and Use of Laboratory Animals as stated by the guidelines of Zhejiang University.

### Synthesis of cationic block copolymers of PEG-PLys

The cationic block polymer of PEG-PLys was synthesized by ring-opening polymerization of monomers of Lys(TFA)-NCA from the ω-NH_2_ terminal group of α-methoxy-ω-amino poly (ethylene glycol) (*M*_w_ = 10 kDa), followed by the removal of the ε-benzyloxy carbonyl protecting groups under treatment with NaOH.

To summarize, 100 mg (10 µmol) of PEG macro-initiator was dissolved in a little amount of dichloromethane, then anhydrous benzene (2 mL) was added, and the mixture was placed in a freeze-drying procedure to remove as much water as possible. Next, the bought Lys(TFA)-NCA reactants and the yielded PEG were moved to the glove box. Separately, the PEG and Lys(TFA)-NCA reactants were dissolved in anhydrous DMF at a concentration of approximately 10 mg/mL. After combining the two DMF solutions indicated before, the reaction was carried out by keeping them at 30 °C for 72 h. The raw PEG-PLys(TFA) product was separated in the refrigerated diethyl ether by precipitation. The precipitate was then moved to an oven for vacuum drying. The produced PEG-PLys(TFA) was dissolved in 1 N NaOH methanol solution overnight at 30 °C to eliminate the protecting groups of ε-benzyloxy carbonyl. It was then transferred three times to sequential dialysis (MWCO: 10 kDa) in 0.01 N-hydrochloric acid and ultra-pure water. Ultimately, lyophilization was used to gather the synthesized PEG-PLys.

### Synthesis of thiolated PEG-PLys [PEG-PLys(SH)

By reacting the primary amino groups in the side chains of the produced PLys segments with 2-iminothiolane, iminothiolane modification was added to PEG-PLys. Initially, 50 mg/mL of PEG-PLys and 25 mg/mL of 2-iminothiolane were individually dissolved in NMP containing 5 wt% LiCl. Additionally, the two solutions were combined for a period of 18 h at 25 °C under stirring and equal molar ratios of lysine and 2-iminothiolane in the presence of DIPEA (10 equiv. relative to the lysine units). The reaction mixture was allowed to precipitate in 60 mL of diethyl ether, which was 15 times more than needed. After collecting and resolving the precipitate in 20 milliliters of 0.01 N HCl, distilled water dialysis was performed (MWCO: 3500). By lyophilization, the final PEG-PLys(SH) was produced. The degree of substitution for each PEG-PLys(SH) was determined according to its ^1^H NMR spectrum, based on the integral intensity ratio of the β-, γ-, and δ-methylene protons of lysine [(CH_2_)_3_, δ: 1.3 to 1.9] to the protons of trim ethylene units of mercaptopropyl groups [HS-(CH_2_)_3_, δ: 2.1 to 2.8]. The calculated substitution degrees of < x > are shown as PEG-PLys(SH_x_), where x stands for the substitution percentage.

### Amplification and purification of plasmid DNA

The plasmid was amplified in the DH5α strain of Escherichia coli bacteria for the current investigation, and it was extracted from the bacterium using an Endo-Free Plasmid Maxi kit in accordance with the manufacturer’s instructions. The ratio of the generated product’s absorbance at 260 and 280 nm was used to calculate its purity (A_260_/A_280_), respectively, using UV spectrophotometry. The index of A_260_/A_280_ used in this study was in the range of 1.8 to 2.0.

### Preparation of PEG-PLys(SH) disulfide crosslinked pDNA condensates

In order to fully cleave the possible disulfide linkage, PEG-PLys(SH) solutions were incubated in DTT-containing aqueous solution (100 mM DTT, 10 mM HEPES buffer, 600 mM NaCl or 0 mM NaCl, pH 7.4) at 25 °C for three hours prior to complexation with pDNA. Additionally, aqueous solutions of pDNA were combined with the previously mentioned PEG-PLys(SH) at different N/P ratios (i.e., the molar ratio of the amine groups (N) in PLys to the phosphate groups (P) in pDNA) to produce pDNA condensates. The thiol groups were oxidized to produce disulfide crosslinking after an overnight incubation at 25 °C with the help of DMSO at 37 °C for 48 h, and then another 48 h of dialysis against HEPES buffer (10 mM, pH 7.4) with the intention of removing the DMSO. The final pDNA concentration of pDNA [pDNA@PEG-PLys(SH)] was adjusted to approximately 33.3 µg/mL for the pDNA condensates unless specifically noted.

### Dynamic light scattering (DLS) measurements

Using Zetasizer Nano ZS90 (Malvern, UK), the formation of the electrostatically formed structures of pDNA@PEG-PLys(SH) was examined. It should be noted that three DLS measurements were made at a temperature of 37 °C and a detection angle of 173°. Additionally, using a cumulant approach, the decay rate relevant to the photon correlation function was examined. In the end, the Stokes-Einstein equation was used to obtain the hydrodynamic diameter.

### Microscopic morphologies by transmission electron microscopy (TEM) measurement

An electron microscope operating at 125 kV acceleration voltages was used to observe TEM images. Using an anion coater, copper TEM grids with carbon-coated collodion film were glow-discharged for 20 s. Uranyl acetate (UA) solution in an identical amount (2% (w/v)) was added to the sample to stain it. For thirty seconds, the grids were immersed in the solution. The excess solution was carefully removed with a filter paper, and the sample grids were allowed to dry in air.

### Tolerability to DNase degradation

The plasmid DNA-containing samples were mixed with a DNase I reaction solution containing all the required components (DNase I: 0.01 units, MgCl_2_: 25 mM, Tris–HCl buffer: 10 mM pH 7.4, pDNA: 33.3 µg/ml) and incubated in presence of heparin (1 mg/mL) at 37 °C. After the predefined reaction periods, saturated EDTA solution was added to terminate the enzymatic reaction, followed by the addition of dithiothreitol (DTT: 100 mM) and saturated anionic dextran sulfate for the release of the plasmid DNA payloads from the self-assembly. After overnight incubation, the reaction solution was collected for qPCR measurements to quantify the intact pDNA.

### Cellular uptake

Cellular uptake efficiencies were quantified based on Flow Cytometry measurement. Herein, Cy5-labeled pDNA was used to prepare a variety of pDNA condensates. C2C12 cells were seeded onto 6-well culture plates (100, 000 cells/well) and incubated overnight in 2.0 mL MCDB131 containing 10% FBS, 10 ng/mL b-FGF and 1% antibiotics in a humidified atmosphere (5% CO_2_, 37 °C). The medium was replaced with the fresh one after 24 h of incubation, followed by addition of a 150 µL pDNA condensate solution (33.3 µg pDNA/mL) into each well. During another 24 h post-incubation at 37 °C, the cells were washed 3 times with PBS with the aim of removing the extracellular Cy5. Ultimately, the cells were detached under treatment with trypsin to create cell suspensions in PBS for subsequent Flow Cytometry measurements.

### In vitro gene expression

C2C12 cells were seeded onto 24-well culture plates (20,000 cells/well) and incubated overnight in MCDB131 (400 µL) containing 10% FBS, 10 ng/mL b-FGF and 1% antibiotics in a humidified atmosphere with 5% CO_2_, at 37 °C. The medium was replaced with 400 µL of fresh medium, followed by the addition of 30 µL of plasmid DNA condensates (1 µg pDNA/well). At 24 h post-incubation, the medium was replaced with 400 µL of fresh medium, followed by another 24 h of incubation. The cells were washed with 400 µL PBS and lysed in 150 µL cell lysis buffer. The luciferase activities of the lysates were determined by quantification of the photoluminescence intensities with aid of a Luciferase Reporter Assay Substrate Kit–Firefly kit.

### Angiogenesis in peripheral ischemia animal model

The peripheral ischemia animal model was established on Bal b/c mice which had undergone hindlimb ischemic surgery. The potential revascularization efficacies were evaluated by means of local dosages (intramuscular injection) of naked pVEGF, and our proposed toroidal virus-like plasmid condensates of pVEGF@PEG-PLys(SH). Briefly, the shipped Bal b/c female mice (4 weeks, weight at 20 ± 2 g) were held for 1 week in an SPF environment; they had been anesthetized with intraperitoneal injections of 5% chloralhydrate solution prior to their surgeries. Their hindlimb ischemic surgeries involved an incision being made into their skin from the medial thigh to the knee and the membranes covering their muscle being carefully dissected. Furthermore, their neurovascular bundles were exposed by piercing the membranous femoral sheath, and the external iliac artery, femoral artery, and peroneal artery, respectively, were separated. Ligations were performed in their proximal and distal femoral arteries with 8/0 PGA fast absorbing sutures and dissections were performed between these two ligations. Finally, the mice were sutured and they received intraperitoneal injections of 10% ampicillin (10 ml/kg). The treated Bal b/c female mice were divided randomly into 3 groups: saline (blank, *n* = 5), naked pVEGF (negative control, *n* = 5), and pVEGF@PEG-PLys (positive, *n* = 5). Pertaining to the treatment groups, 0.1 mL solutions containing 10 µg pVEGF were locally injected into the mice. The vasculatures in their hindlimb were monitored for up to 28 days.

### Immunofluorescence for the identification of VEGF expression and neo-vasculature

For immunofluorescence staining, muscular tissues from the mouse ischemic limbs were cryo-sliced into 10 μm thick sections and stained with antibodies specifically targeting VEGF-A and CD 31, with the aim of identifying the expressed VEGF and potential neo-vasculature. Briefly, the freshly prepared tissue cryo-sections were sequentially incubated for 1 h. with VEGF-A and CD31 antibodies. Note that the aforementioned primary monoclonal antibodies subsequently reacted with the secondary antibodies conjugated with Cy3 (the primary antibody of VEGFA) and FITC (the primary antibody of CD31). Eventually, the immunostained cyroslices were transferred into CLSM measurements.

## Results and discussion

### Synthesis of block copolymeric PEG-PLys(SH)

**Fig. 1:**
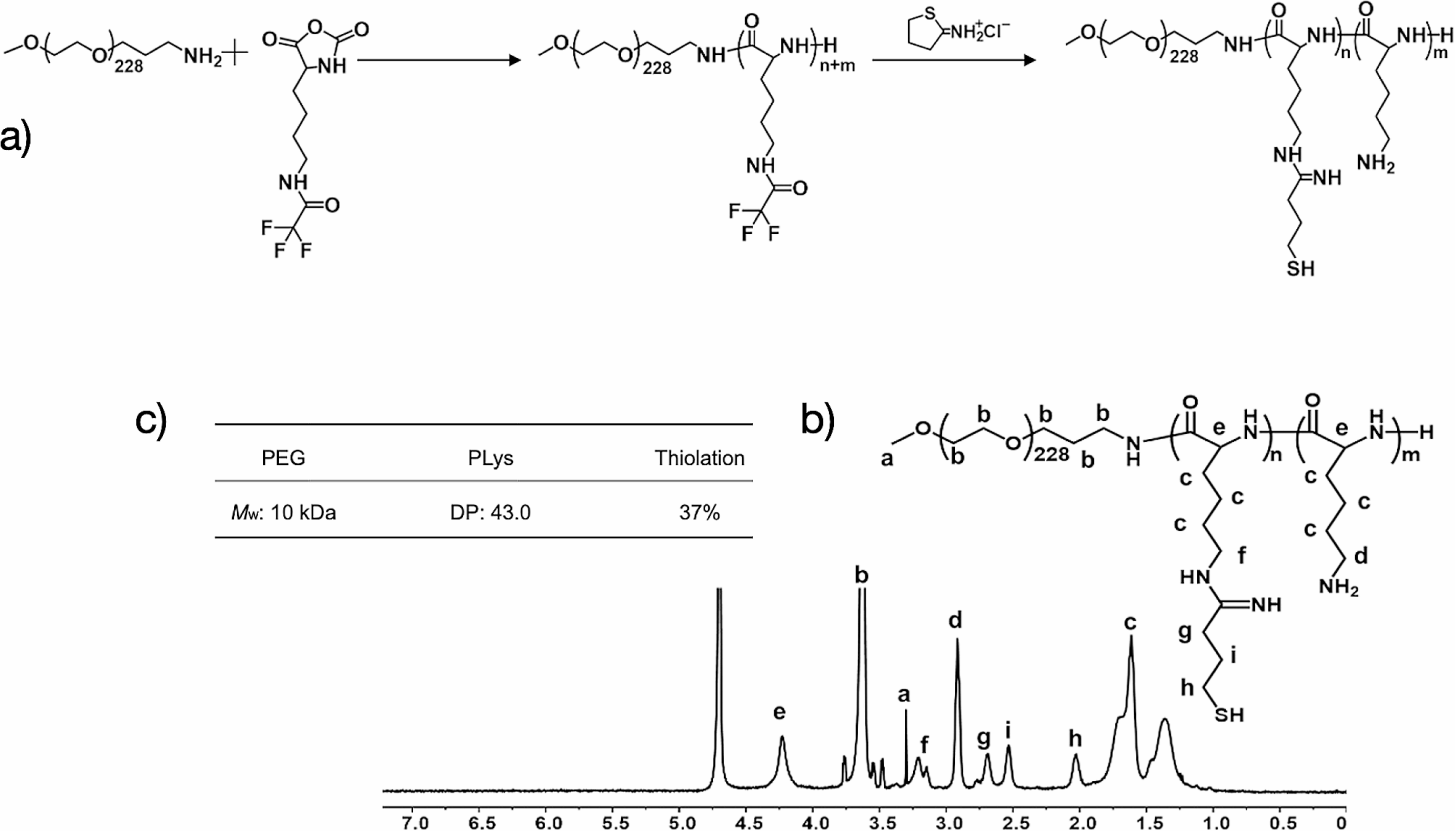
Synthetic route and ^1^H NMR spectrum of block copolymeric PEG-PLys(SH). (**a**) Synthetic route of the thiolated block copolycationic PEG-PLys(SH); (**b**) ^1^H-NMR spectrum of the yielded block copolymer of PEG-PLys(SH) in D_2_O; (**c**) Chemical descriptions of the yielded PEG-PLys(SH)

The synthetic procedure shown in Fig. 1a was followed in order to produce the polymeric PEG-PLys(SH) copolymer. In short, the methoxy-poly(ethylene glycol)-amine (Meo-PEG-NH_2_, 10 kDa) as linear macro-initiator was used to initiate the ring-opening polymerization of the Lys(TFA)-NCA monomers, which resulted in the synthesis of the parental block copolymer of PEG-PLys. Following the deprotection of the TFA residues in the synthesized PLys (TFA) segments, the block copolycationic PEG-PLys was produced by alkaline hydrolysis. It should be noted that proton nuclear magnetic resonance spectroscopy (^1^H-NMR, Bruker Avance II 400, SUI) was used to determine that the synthesized PLys segments had a polymerization degree of approximately 43.0. Additionally, the ring-opening reaction between the primary amines in the lysine residues of PEG-PLys and the cyclic imidoester 2-iminothiolane was intended to produce the final PEG-PLys(SH) from the amidine-linked mercaptopropyl moieties. The transformation of the primary amines into amidines was verified by observing the ^1^H NMR spectra of PEG-PLys (Fig. S1) and PEG-PLys(SH) (Fig. 1b). This was demonstrated by an apparent downfield shift in the ε-methylene protons of the lysine residues (e to f, Fig. 1b) and the appearance of the proton signals associated with the CH_2_ units of the γ-mercaptopropyl groups (g and i, Fig. 1b). The degree of substitution was calculated from these characteristic signals (detailed in the Materials and Methods section) and the substitution percentage of 2-iminothiolane relative to the total primary amines in PLys was estimated to be approximately 37% (Fig. 1c).

### Fabrication of virus-like pDNA condensates

With the aim of achieving progressive VEGF expression, the VEGF-encoding pDNA was requested to translocate across the cytomembrane into the cell interiors. Nonetheless, the negatively charged and microscaled pDNA macromolecules could not penetrate the lipophilic bilayer of the cytomembrane. Moreover, the ubiquitous nucleases in the extracellular environment caused the rapid degradation of the pDNA. To this end, the neutralization of pDNA into nanoscaled DNA condensates was incited to protect the pDNA from nuclease digestion and its payloads from being transported transcellularly. Note that the natural virus employs an “intelligent” transportation approach to circumvent sequential biological barriers and accomplish a variety of requested functionalities [24–26]. In particularly, its macromolecular genomic materials are packaged in an orderly fashion into circular condensates beneath multiple segregated surroundings (e.g. its capsid, envelope). Nonetheless, virus’ inherently detrimental immunogenicity and its genome’s propensity to integrate into the host chromosomes encourages the development of intelligent non-viral gene delivery carriers highly availability for gene therapy with clinical applications.

With the aim of mimicking a natural virus, we proposed that the pDNA be packaged by oppositely charged block copolymers composed of a nonionic hydrophilic segment (PEG) and a cationic segment (PLys). The polyionic complexing process between pDNA and PLys segments was expected to cause spontaneous structural ordering of the rigidly supercoiled pDNA (persistence length: 50 nm in a physiological environment) into core-shelled architectural pDNA condensates. Namely, the ion-complexed pDNA condensate with polylysine was surrounded by a dense hydrophilic PEG shell, which enable single molecular pDNA condensation due to the spatial passivation of PEG.

Note that an essential aspect of DNA condensation is the scheme by which the rigid supercoiled giant DNA molecule is packaged. Our pioneer research has revealed that selectively spooling single molecular pDNA into a nanosized toroidal structure or folding it into a rod-like structure is accomplished by polyionic complexing block catiomers into pDNA condensates in varying NaCl concentrations [19, 20]. The interactive potency between the pDNA and block catiomers was determined to play a critical role in defining the ultimate structure of the pDNA molecule, and the formation of toroidal or rod-like structures was found to be achieved by complexation in 600 or 0 mM NaCl solutions, respectively. Compared with the rod-like condensates, the toroidal condensates possess appeared to exert superior biological functions capable not only of elevating in vitro transcription but also of elevating in vivo gene transduction efficiencies. These results indicate the great utility of the toroidal pDNA packaging as an intriguing tool for gene therapy.

Hence, an attempt was made to complex the block copolymeric PEG-PLys(SH) together with pDNA into toroidal condensates as gene delivery systems. Note that our previous studies have demonstrated markedly higher transcription activities of toroidal pDNA condensates or even rod-like pDNA condensates (significantly higher than naked supercoiled pDNA) in cell-free transcription system [27]. This extraordinary result implied our proposed orderly condensed pDNA delivery system can directly elicit gene expression, irrespective of DNA release. However, aiming to stabilize the unique toroidal pDNA condensates, particularly avoiding the potential structural disassembly in the extracellular compartment, we attempted to introduce thiol groups into the side chain of PLys segments of PEG-PLys, where crosslinkage of the complex compartment could be achieved though inter- or intramolecular disulfide linkage between the thiol residues of PLys(SH). This crosslinking strategy is believed to markedly enhance the colloidal stability within the biological environment, particularly reducing the possibility of dissociation as a consequence of electrostatic exchange reactions with the charged species in the biological environment, such as in glycosaminoglycans.

Herein, the toroidal plasmid condensates were prepared by mixing PEG-PLys(SH) copolymer with pDNA in the presence of DTT (preventing the primary disulfide crosslinkages from forming prior to electrostatic complexation) and NaCl (0 mM or 600 mM) at varied N/P ratios (defined as the molar ratio of the amine groups from the polylysine segments in the block copolymer and the phosphate groups from the pDNA). As shown in Fig. S2, charge neutralization of the negatively charged pDNA appeared to be achieved in presence of cationic PEG-PLys(SH) starting at N/P ratio of 1. In the present study, pDNA condensates by PEG-PLys(SH) at N/P ratio of 2 (ensuring the complete charge neutralization) was chosen for the subsequent experiments.

**Fig. 2:**
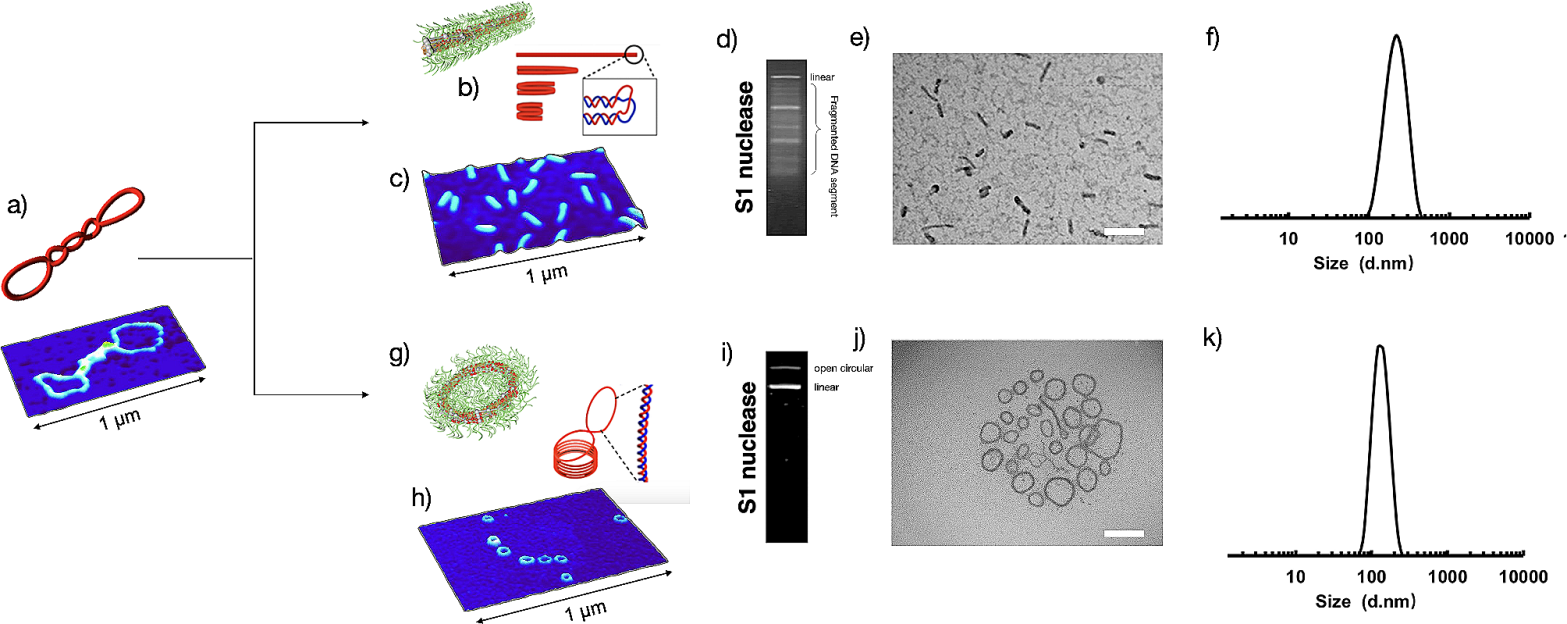
pDNA condensates investigated by AFM, TEM, DLS measurements and enzymatic reactions in presence of S1 nuclease for insights into the dissociations of dsDNA into ssDNA in pertinent to pDNA condensation. (**a**) Supercoil pDNA and AFM morphology; (**b**) The proposed pDNA condensation pathway for rod-like condensates; (**c**) AFM measurements for rod-like condensates; (**d**) S1 nuclease assay for insight into the dissociations of dsDNA into ssDNA for rod-like condensates; (**e**) TEM measurements for rod-like condensates, scale bar: 200 nm. Note that the frequency of the rod-like structures were quantitively determined to be approximately 95.4%; (**f**) DLS measurements for rod-like condensates (intensity-based); (**g**) The proposed pDNA condensation pathway for toroidal condensates; (**h**) AFM measurements for toroidal condensates; (**i**) S1 nuclease assay for insight into the dissociations of dsDNA into ssDNA for toroidal condensates; Note that the frequency of the toroidal structures were quantitively determined to be approximately 89.6%; (**j**) TEM measurements for toroidal condensates, scale bar: 200 nm; (**k**) DLS measurements for toroidal condensates (intensity-based)

Furthermore, the microscopic morphologies of the yielded pDNA condensates were investigated by Atom Force microscopy (AFM) and transmission electron microscopy (TEM) measurements. As shown in Fig. 2a, the naked pDNA was determined to have distinctive supercoiled conformation, possessing micrometer scale. Yet, pDNA upon complexation with PEG-PLys(SH) was observed to undergo marked condensation into nanoscaled structures. For instance, pDNA@PEG-PLys(SH) at NaCl (0 mM) was observed to form uniform rod-like morphologies (referred to as rod-like condensates, Fig. 2c and e), as opposed to the uniform donut-like toroidal morphologies at NaCl (600 nM, referred to as toroidal condensates, Fig. 2h and j). As clarified by our previous researches, the rod-like condensates were obtained by means of the regular folding of dsDNA strands into DNA bundles (with dsDNA being dissociation into ssDNA in the folding ends, Fig. 2b), yet the toroidal condensates were obtained through dsDNA self-spooling (characterized by intact dsDNA in the toroidal pDNA condensates, Fig. 2g) [17, 18]. The self-spooling pathway for DNA condensation was indicated by an S1 nuclease assay (Fig. 2d and i), wherein the toroidal pDNA condensates was incubated in presence of S1 nuclease (capable of specifically cleaving ssDNA). Indeed, single band corresponding to the intact pDNA was confirmed for the toroidal condensates (Fig. 2j), as opposed to fragmentation for rod-like condensates (Fig. 2d). Note that the observed fragmented DNA was attributed to the dissociation of double-strand DNA into single-strand DNA at the folding ends of the rod-like condensates. Hence, the unique self-spooling DNA condensation process of the toroidal condensates is postulated to be advantageous for the subsequent transcription rather than abrupt turning for rod-like condensates. Of note, the natural virus adopted this self-spooling pattern in pertinent to the packaging of DNA cargo in capsid. Moreover, the nucleosome, a complex formed between individual DNA and a group of histones, was characterized to possess DNA-spooling motif. Hence, the toroidal condensates were utilized for our proposed revascularization therapy in treatment of hindlimb ischemia. Furthermore, consistent with TEM measurements, dynamic light scattering intensity (DLS) measurement revealed that the hydrodynamic diameters (intensity-based) of rod-like and toroidal condensates were approximately 177 nm and 186 nm, respectively (Fig. 2f and k).

### Disulfide crosslinked plasmid condensates for their persistent retention of the intact pDNA payloads in extracellular compartments and the ready liberation of plasmid payloads in the cell interiors

**Fig. 3:**
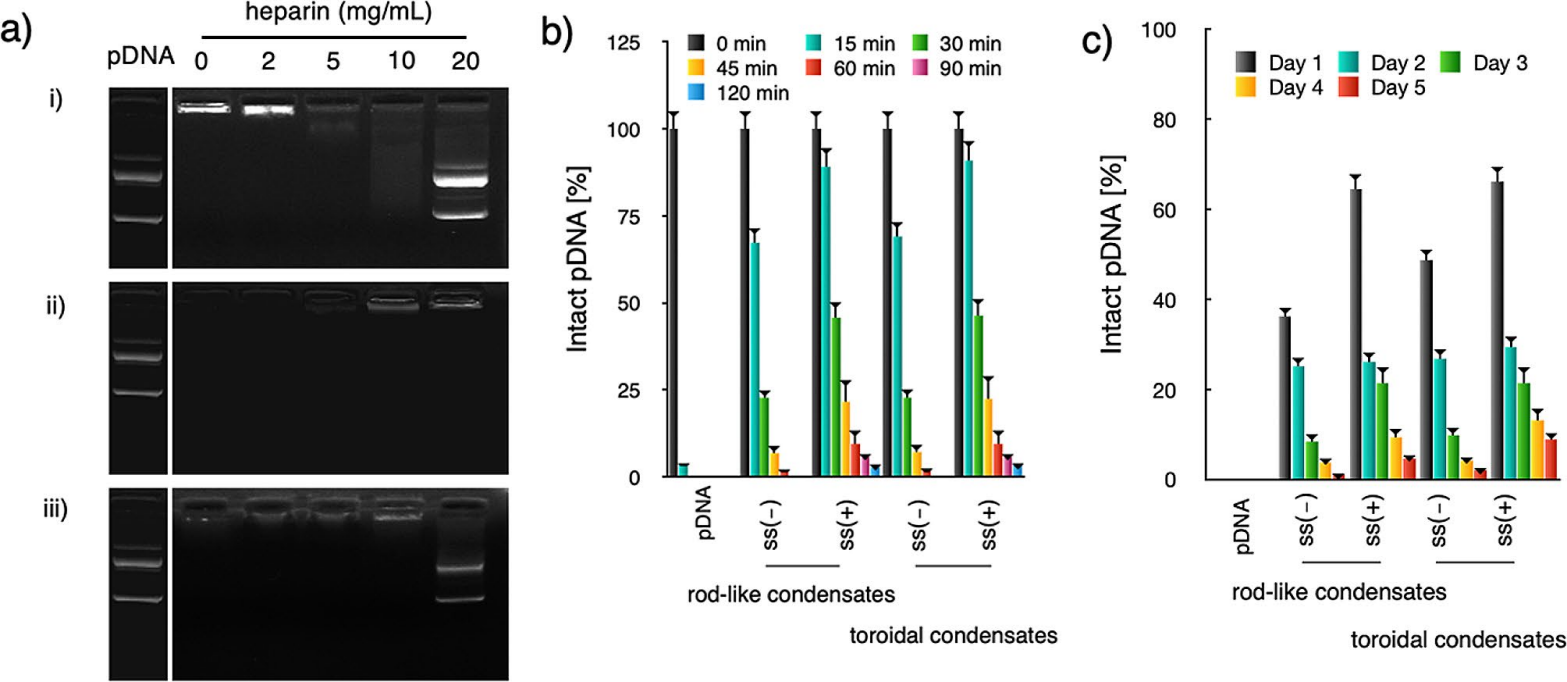
Structural stabilities and tolerabilities in the physiological environment of the proposed toroidal pDNA condensates. **a**) Assessment of structural stabilities of toroidal pDNA condensates under treatment of heparin at varied concentrations. Upper lanes: toroidal pDNA condensates from PEG-PLys; Middle lanes: toroidal pDNA condensates with disulfide crosslinking from PEG-PLys(SH) in absence of GSH (10 µM, mimicking the extracellular microenvironment); Lower lanes: toroidal pDNA condensates from PEG-PLys(SH) in presence of GSH (10 mM, mimicking the intracellular microenvironment); (**b**) In vitro tolerabilities of pDNA condensates from PEG-PLys and PEG-PLys(SH) under incubation in presence of 0.01 U DNase I and 1.0 mg/mL heparin. Note that ss(-) and ss(+) have *p* value < 0.05 from ANOVA analysis for both rod-like condensates and toroidal condensates. The data were represented as the mean ± standard deviations (s.d.) (*n* = 5); (**c**) In vivo tolerabilities of pDNA condensates from PEG-PLys and PEG-PLys(SH) upon intramuscular injection, wherein the levels of the intact pDNA were quantified by qRT-PCR measurements. Note that ss(-) and ss(+) have *p* value < 0.05 from ANOVA analysis for both rod-like condensates and toroidal condensates. The data were represented as the mean ± standard error of the mean (s.e.m.) (*n* = 5)

In order to prevent premature dissociation in the harsh physiological environment, the virus-like toroidal pDNA condensates were designed with redox-responsive crosslinking to withstand potential exchange reactions and attain adequate colloidal stability in the environment. Specifically, in the presence of bio-existing polyanionic glycosaminoglycan of heparin, pDNA condensates lacking disulfide crosslinkages could be expected to structurally dissociate readily and release the pDNA payloads; on the other hand, pDNA condensates with disulfide crosslinking could be expected to have improved colloidal stability. In order to optimize the transcriptional and translational machinery that follows, it is crucial that the suggested pDNA condensates not only have sufficient stability in the extracellular environment but also have the ability to cleave the disulfide crosslinking network. This allows transcriptase to slide along DNA strands. Our suggested disulfide bond is particularly interesting in relation to the reversible crosslinking between the extracellular and intracellular compartments because it is easily cleaved into free thiols in response to the intracellular reducing environment. Considering that the interior of the cell is characterized as a highly reducing environment committed to the removal of harmfully reactive oxygen species, abundantly reducing glutathione (GSH) substances have been determined in the cytosol (in the several 10–100 mM range), but the extracellular level of GSH was estimated to be in the 10–100 µM range. As a result, it is speculated that the redox-responsive disulfide linkaging in the manufactured nanocolloidals will selectively cleave in the cytosol, whilst the disulfide crosslinking will remain relatively stable in the extracellular milieu (which includes the bloodstream and extracellular matrix). Because of the variety of anionic polyanions found in the extracellular compartment and cytoplasmic structures (such as glycosaminoglycans), the electrostatically-assembled nanocolloidals without covalent crosslinkages are therefore vulnerable to exchange reactions with polyanionic species. Consistent with our speculation, ready structural disassembly so as for pDNA release was confirmed for the pDNA condensates from nanocolloidals void of crosslinking (Fig. 3a, i) in presence of extracellular-enriched glycosaminoglycans (heparin, releasing starting from heparin 5 mg/mL). In contrast, preserved structure was determined for the pDNA condensates from nanocolloidals with crosslinking despite incubation with high concentration of heparin (20 mg/mL) (Fig. 3a, ii). Furthermore, disulfide crosslinking was implied to be cleaved, as evidenced upon incubation at GSH (10 mM, mimicking intracellular redox environment) in presence of heparin (20 mg/mL) (Fig. 3a, iii). To this end, facile redox-responsive crosslinking was confirmed for our proposed disulfide-functionalized pDNA condensates, which linkages endow adequate colloidal stability in the extracellular environment but cleavable in the targeted intracellular compartments, thereby being conjectured to facilitate progressive transcellular gene transportation and gene expression in the targeted cells.

In addition, the fatal threat to impede DNA delivery is the vulnerable nature of DNA to nuclease degradation. The enhanced colloidal stabilities due to disulfide crosslinking is envisioned to prevent the premature liberation of the encapsulated pDNA payloads from enzymatic digestion in the physiological environment (e.g. DNase I: 0.01 U/mL in the bloodstream). Herein, toroidal pDNA condensate from PEG-PLys and PEG-PLys(SH) was incubated in the presence of heparin (10.0 mg/mL) and DNase I (0.01 U/mL) with the aim of mimicking the physiological microenvironment. The remaining intact pDNA upon varied incubation periods was quantified by qRT-PCR measurement. As shown in Fig. 3b, naked pDNA was rapidly digested within 30 min. Yet, markedly enhanced protection of the encapsulated pDNA was confirmed by the toroidal condensates. Approximately 24% intact pDNA was observed to remain at 30 min post incubation for the toroidal condensates from PEG-PLys. Moreover, disulfide crosslinking appeared to conduce to significantly prolonged survival of the encapsulated DNA in heparin and DNase I-enriched extracellular environment. Markedly intact pDNA was determined to remain despite extended incubation (120 min). Furthermore, a variety of pDNA condensates were administered through intramuscular dosage. The intact pDNA in the dosed muscular tissues were quantified by qRT-PCR measurements. As shown in Fig. 3c, negligible intact pDNA could be detected at the dosed sites for the naked pDNA, which should be attributable to the ready degradation of pDNA by the enriched nucleases in the physiologic environments. Moreover, the inherent immunogenicity of pDNA was also believed to be capable of stimulating the immune responses so as for immune clearance of the exogenous nucleic acids. On the contrary, consistent retention of the intact pDNA was determined for pDNA condensates with PEG-PLys or PEG-PLys(SH). The plausible reason for this improved retention should be attributable to the exteriors of pDNA condensates, particularly the crowded PEG surroundings, which could not only substantially reduce the accessibilities of the environmental nucleases towards the interior pDNA payloads but also present as the spatial barriers to prevent recognition from immune systems. Of note, approximately 200-fold intact pDNA was estimated for pDNA condensates on day 1 post intramuscular dosage in relative to the naked pDNA, approximately 300-fold on day 5. In consistent with the in vitro tolerabilities in treatment with DNase I, pDNA condensates with disulfide crosslinking exhibited relatively improved retention at the dosage sites as compared to those lacking disulfide crosslinking upon intramuscular dosage. Most likely, pDNA condensates with disulfide crosslinking could substantially prevent structural disassembly from releasing the internal pDNA payloads in the complicated biological environment, thereby accounting for the limited nuclease degradation or clearance of the exogenous nucleic acids. To this end, the well-defined exteriors for accommodation of pDNA cargo are imperative for the pDNA therapeutics to purse improved bioavailabilities and adequate gene therapy outcomes.

### Persistent gene expression by virus-like toroidal plasmid condensates

**Fig. 4:**
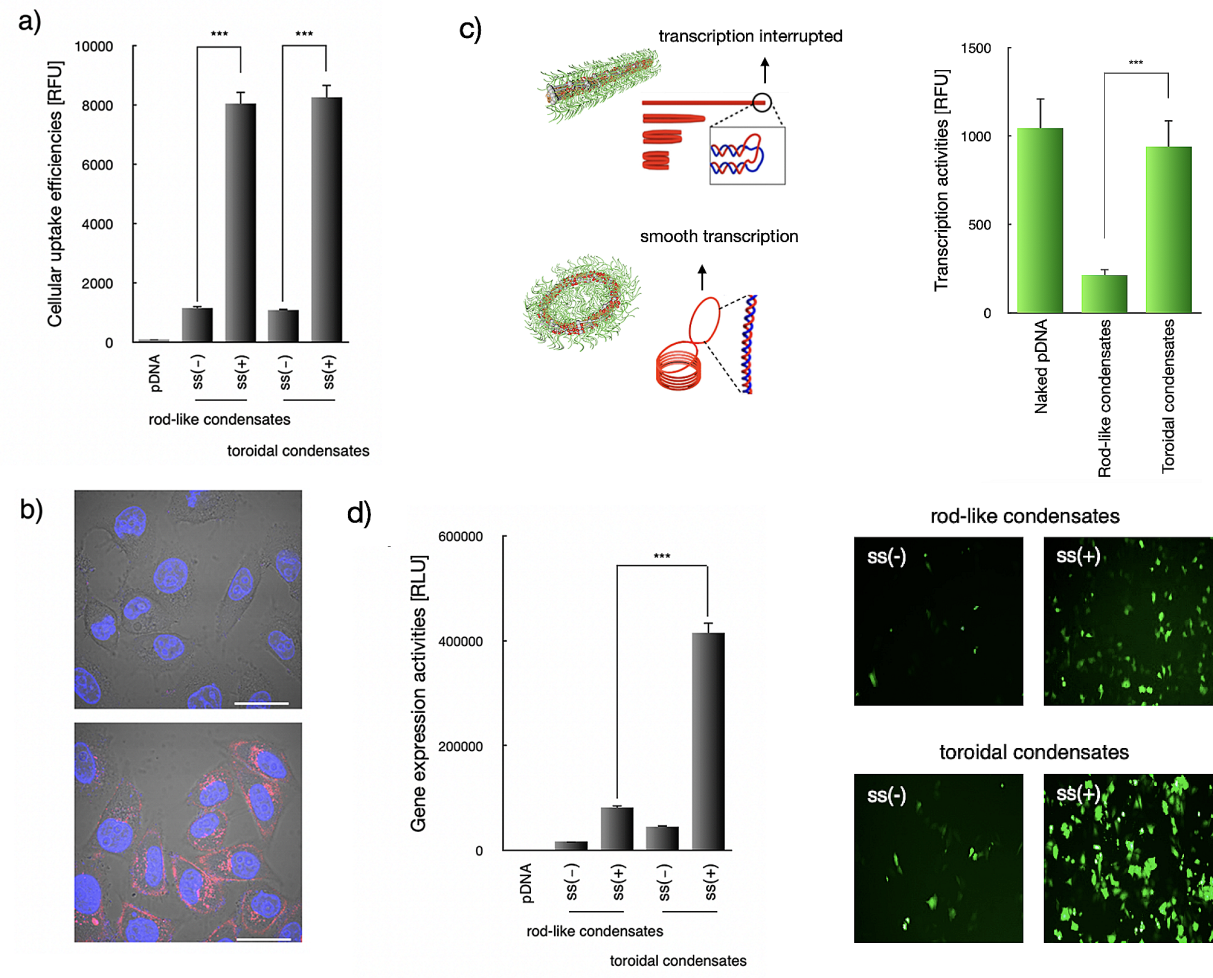
Cellular uptake and gene expression efficiencies of rod-like and toroidal condensates with [ss(+)] and without disulfide crosslinking [ss(-)]. (**a**) Quantification of cellular uptake efficiencies by Flow Cytometry, wherein pDNA was labeled by Cy5 for preparation of pDNA condensates that were incubated for 48 h in C2C12 cells. (^***^p < 0.001, student t test). The data were represented as the mean ± standard deviations (s.d.) (*n* = 3); (**b**) Intracellular distributions of pDNA by CLSM at 24 h post-incubation. Upper: pDNA; Lower: toroidal pDNA condensates. Red: pDNA, blue: nuclei. Scale bar: 20 μm; (**c**) Cell-free gene transcription activities of naked pDNA, rod-like and toroidal condensates. The data were represented as the mean ± standard deviations (s.d.) (*n* = 3); (**d**) Expression efficiencies of Luc in C2C12 cells by rod-like and toroidal pLuc condensates with or without disulfide crosslinking (^***^p < 0.001, student t test). The data were represented as the mean ± standard deviations (s.d.) (*n* = 4)

The cellular uptake and gene expression efficiencies of virus-like toroidal plasmid condensates were evaluated in C2C12 cells. Consistent with our speculation, negligible cellular uptake was confirmed for the naked pDNA (Fig. 4a). However, pDNA condensation appeared to facilitate pDNA internalization into cells, as evidenced by the marked intracellular levels of pDNA in the cell interiors. In addition, pDNA condensates with disulfide crosslinkages (both rod-like and toroidal condensates) were determined to facilitate higher degree of cellular uptake efficiency of approximate 8.1-fold relative to the pDNA condensates devoid of disulfide crosslinking (Fig. 4a). This enhancement was considered to be as a consequence of the improved structural stability due to disulfide crosslinking, thereby enabling progressive cell internalization of the nanoscaled pDNA condensates. Moreover, the cellular uptake efficiencies of the rod-like and toroidal condensates were considered nearly comparable. On the other hand, confocal laser scanning microscopy measurement captured consistent results by which efficient pDNA internalization into cell interiors was confirmed (Fig. 4b), in contrast to the minimal intracellular level of pDNA for naked pDNA (as comparable to the background).

As demonstrated above, the rod-like pDNA condensates were interpreted as the regular folding of the DNA strands into the DNA bundles. Note that the lateral packing into the DNA bundle was accomplished by unwinding of dsDNA into ssDNA at the ends of the rod-like condensates. In contrast, toroidal condensates were elucidated to be packed by following consistent self-spooling process without compromise of unwinding of dsDNA. Therefore, the intriguing self-spooling DNA condensation process of the toroidal condensates is conjectured to be most favorable in pertinent to the transcription activities with respect to the potential progressive looping in synthesis of mRNA as opposed to the abrupt turning for rod-like condensates. In consistent with our speculations, the toroidal condensates elicited comparable high transcription activities in relative to the naked pDNA, approximately 2.7-fold higher than rod-like condensates.

Consistent with the cellular uptake efficiencies and transcription activities, the gene expression efficiencies appeared to follow similar trends (Fig. 4c). Herein, pDNA-encoding luciferase as the reporter gene was employed as the payload for the quantification of the gene expression efficiencies of the proposed pDNA condensates. Negligible gene expression was confirmed for naked pDNA, and the gene expression levels of the pDNA condensates devoid of disulfide crosslinkages were determined to be limited (nearly comparable to the background). Nonetheless, marked gene expression was confirmed for the pDNA condensates with disulfide crosslinking. Of interest, significantly higher gene expression was obtained from toroidal than rod-like condensates, approximate 4.8-fold (Fig. 4c). In view of the comparable cellular uptake efficiencies of toroidal and rod-like condensates, the superior gene expression by the toroidal condensates could be attributed to their facilitated transcription activities. Given that the transcription machinery slides along the DNA during the transcription process, it can be reasonable to assume that the process may continuously proceed in the toroid structures along an infinite loop of DNA. Alternatively, in the rod-like structures, the transcriptional process might proceed along the rod axis but be interfered at the rod end because of the impaired integrity of the double stranded structure of DNA. These feasible mechanisms involved in the transcription process are consistent with the observation that toroidal condensates exert significantly higher gene expression efficiencies than rod-like condensates. In addition, persistent gene expression was also confirmed. Herein, in vitro 3D cell spheroids were used for real-time measurement of gene expression profile of our proposed pDNA condensates, wherein pDNA-encoding the secreting Gaussia luciferase was used, therefore allowing real-time measurement (Kronos) of luciferase expression by measuring luminescence intensities when the substrate (luciferin) added in the culture medium [28]. As shown in Fig. S3, the nearly constant gene expression of the pLUC payloads in up to 12 days (Fig. S3), which is believed to be important for subsequent angiogenesis when pVEGF was used.

### Revascularization in the in vivo treatment of peripheral ischemia

**Fig. 5:**
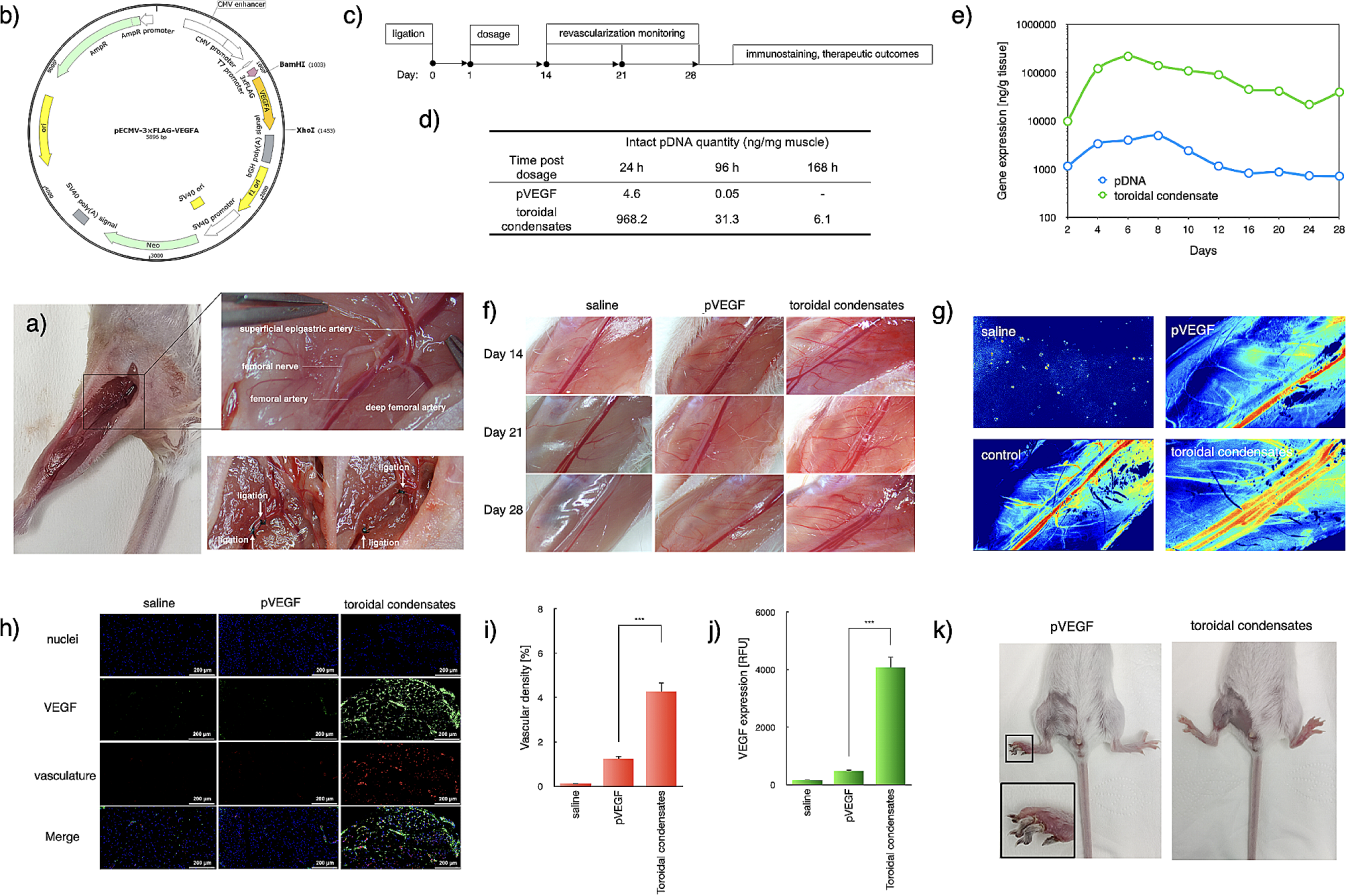
Revascularization in hindlimbs by local dosage of toroidal condensates encapsulating pVEGF. (**a**) Anatomy of the established hindlimb ischemia model. Ligations were made in the femoral artery at the proximal and distal site; (**b**) Genomic sequence of VEGF-expressing pDNA (10 µg) for treatment of hindlimb ischemia; (**c**) Therapeutic scheme; (**d**) Quantitative measurement in pertinent to the retention of the dosed pDNA post intramuscular administration by RT-PCR measurement; (**e**) Time-dependent measurement for in vivo gene expression; (**f**) Angiogenesis in mouse hindlimbs post ligation; (**g**) Visualization of blood flow by Laser Speckle Flowgraphy; (**h**) Immunostaining for insights into the VEGF expression and the consequent neo-vasculature formation in ischemic hindlimbs through local dosage of saline, naked pVEGF, and toroidal pVEGF condensates on Day 28 post-dosage. Red: vasculature, blue: cell nuclei, green: VEGF; (**i**) Quantification of VEGF expression. The data were represented as the mean ± standard deviations (s.d.) (*n* = 5). (^***^p < 0.001, student t test); (**j**) Estimation of vascular densities based on quantification of CD31-positive pixels, wherein the vasculatures were stained by anti-CD31 antibody. The data were represented as the mean ± standard deviations (s.d.) (*n* = 5). (^***^p < 0.001, student t test); (**k**) Therapeutic outcomes on day 28 post ligation

The schematic illustration of hindlimb ischemia was established on Bal b/c mice by means of hindlimb ischemia surgery (Fig. 5a). Briefly, an incision was made in the skin from the medial thigh to the knee and the membranes covering the muscle were carefully dissected. Furthermore, the neurovascular bundle was exposed by piercing the membranous femoral sheath, and the external iliac artery, femoral artery, and peroneal artery, respectively, were separated. Ligations were performed in the proximal and distal femoral arteries with 8/0 PGA fast absorbing sutures and a dissection was made between the two ligations. Eventually, the mice were sutured and scheduled for intraperitoneal injections of 10% ampicillin (10 mL/kg). The potential revascularization efficacies were evaluated by means of local dosage (intramuscular injection) of the naked pVEGF (genomic sequence in Fig. 5b and Fig. S4) and our proposed toroidal virus-like pVEGF condensates (10 µg pVEGF in 0.1 mL PBS) were evaluated from 24 h to 28 days post-suture (Fig. 5c). In view of several pioneering clinical gene therapy pipelines directly using the naked pDNA for expression of growth factors in seeking regenerative medicinal outcomes, e.g. Collategene (HGF-encoding pDNA, AnGes) [29] and NL003 (HGF-encoding pDNA, clinical phase III, Northland Co.) [30], the naked pDNA was also included as in the study. To start up, the local retention of pDNA and in vivo gene expression was estimated by RT-PCR measurement and ELISA measurement, respectively, in pertinent to the dissected muscles at the dosage sites. As shown in Fig. 5d, the naked pDNA appeared to be susceptible to rapid degradation, which should be attributable to the enriched nucleases (e.g. DNase I) in the extracellular compartments. On the contrary, 2-3-fold higher level of the intact pDNA was confirmed once pDNA encapsulated into PEG-PLys(SH)-based toroidal condensates, approving the appreciable protecting properties by our strategic manufacture of core-shell delivery systems. In addition, toroidal condensates exerted drastically higher gene expression activities post local intramuscular administration (Fig. 5e), due to improved survival in nuclease-enriched extracellular microenvironment and promoted transcellular trafficking by our proposed scaled-down condensation into nanoscaled delivery vehicles. Of note, persistent expression and retention of VEGF exceeding 4 weeks was determined, which is believed to be favorable to achieve successful regeneration of neo-vasculature in the diseased regions.

Eventually, therapeutic outcomes were evaluated in our established hindlimb ischemia model. As shown in Fig. 5f, the blood vessels were observed to gradually disappear post-ligation. In particular, almost no recognizable micro-blood vessels were seen on day 28 post-ligation (Fig. 5f). Meanwhile, Laser Speckle Flowgraphy measurement indicated minimal blood flow post-ligation (Fig. 5g). Consistently, immunostaining (CD31) could barely identify the vascular endothelial cells in the vascular section from the hindlimb tissues (Fig. 5h and i). Moreover, the natural endogenous VEGF level was also determined to be minimal (Fig. 5j). In addition, the therapeutic outcomes by dosage of the naked pVEGF were also determined to be limited, as evidenced by the limited neo-vasculature and necrosis in the extremities (Fig. 5k). The negligible neo-vasculature by the naked pVEGF can be explained by its low expression of the angiogenic VEGF (Fig. 5h and j). The significant in vivo gene expression, despite low, is speculated to due to the fluid pressure caused by intramuscular dosage, which could physically lead to improved permeability of the cytomembranes [31]. Most likely, the temporal loss of the intactness of cytomembrane allows the possible translocation of pDNA into the cell interior despite its large scale. To this respect, clinical trials, including Collategene using the naked pDNA could achieve significant gene expression efficiencies and positive therapeutic outcomes despite markedly higher dosage required.

In contrast, our proposed toroidal pVEGF condensates appeared to induce high levels of angiogenic VEGF expression (Fig. 5h and j), thereby inducing the revascularization in the VEGF-enriched regions (Fig. 5f, and h). In particular, a multiple of new femoral arteries were clearly observed to form in parallel with the pre-existing the ligated femoral artery. Moreover, the micro-vessels and capillaries originated from these femoral arteries elicited spread of the neo-vasculature formation to the entire hindlimb (Fig. 5e and f), consequently contributing to abundant blood flow in the vasculature-regenerated tissues (Fig. 5g). Accordingly, it is not surprising that the lack of vasculature in the hindlimb in supplying the fundamental oxygen and nutrients could lead to necrosis in the extremities for the naked pVEGF group. On the contrary, ischemia was relieved as indicated by the apparent absence of necrosis in the hindlimb under treatment of the toroidal pVEGF condensates (Fig. 5k).

### Excellent safety profile of toroidal condensates

**Fig. 6:**
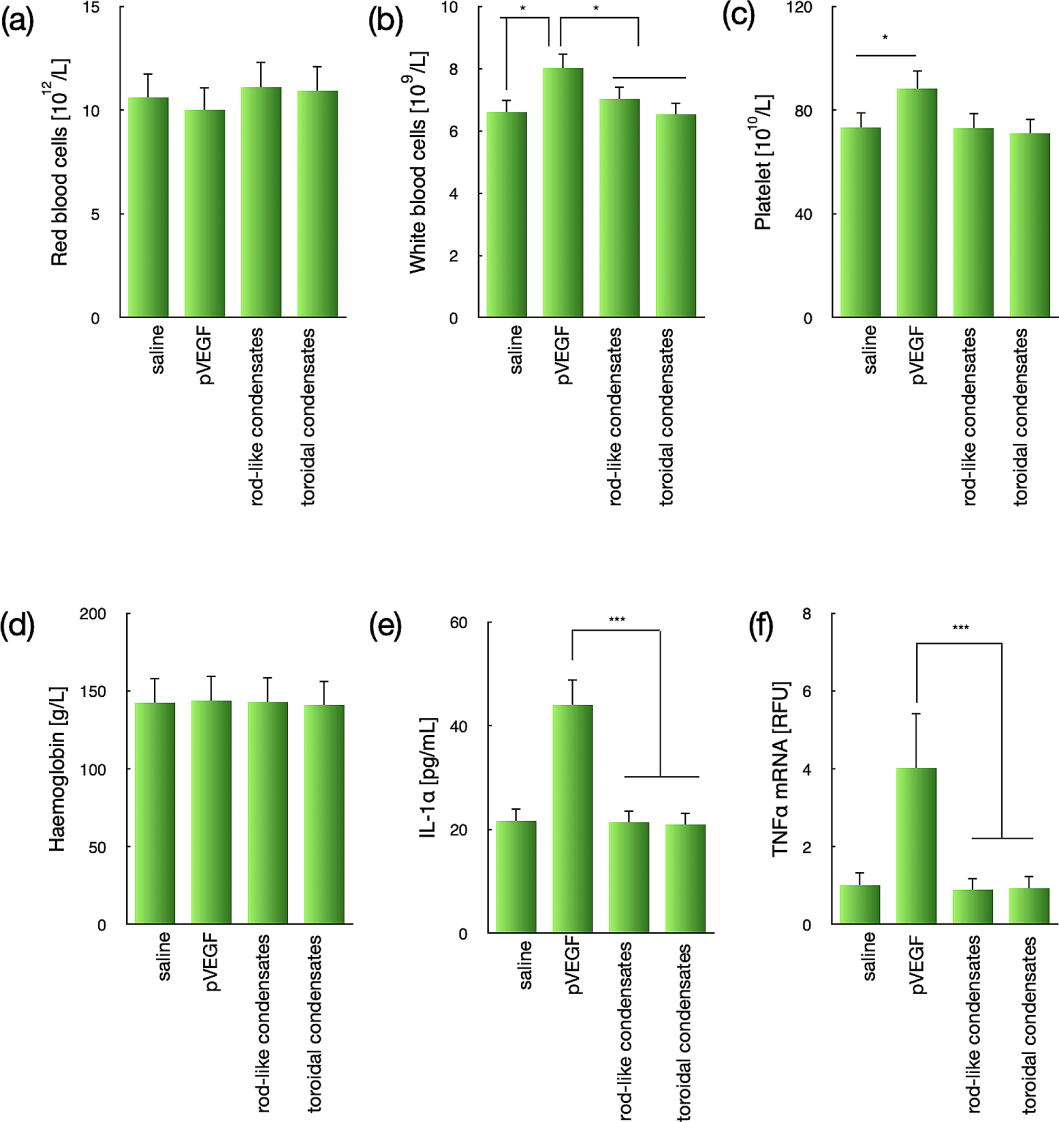
Safety profiles of the proposed toroidal condensates at 24 h post intramuscular injection. (**a**) red blood cells; (**b**) white blood cells; (**c**) platelet; (**d**) haemoglobin; (**e**) IL-1α; (**f**) THF α. The data were represented as the mean ± standard deviations (s.d.) (*n* = 5). (^***^p < 0.01, ^***^p < 0.001, student t test)

Eventually, the safety profiles of our proposed DNA condensates were examined at 24 post intratumor injection. The blood samples were collected , followed by sedimentation to obtain serum for quantification of the immunostimulatory cytokines. As shown in Fig. 6, no significant changes were determined for the DNA condensates, either rod-like condensates or toroidal condensates, in pertinent to red blood cells (Fig. 6a), white blood cells (Fig. 6b) and platelets (Fig. 6c). Meanwhile, quantification of haemoglobins (Fig. 6d) and immunostimulatory cytokines (including IL-1α and TNF-α) (Fig. 6e and f) for the DNA condensates-treated mice was also determined to be comparable to the saline control, again indicating the appreciable biocompatibilities of the proposed PEGylated DNA condensates. Nonetheless, the naked pDNA was observed to elicit significantly elevated levels of immunostimulatory cytokines, which should be attributable to the immunogenicity of pDNA due to direct exposure in the extracellular environment. For instance, the exposure of the naked DNA is the molecular basis for progression of rheumatoid arthritis [32]. Hence, a number of strategies using cationic materials for complexation of the naked DNA have demonstrated the alleviation of pro-inflammatory responses in rheumatoid arthritis [33]. To this respect, our proposed pDNA condensates with additional PEG shielding could reduce the exposure of pDNA as antigen. Apparently, the elicited immunostimulatory from the use of the naked pDNA were unfavorable from viewpoint of regenerative purpose. Therefore, PEGylated DNA condensates could substantially minimize the unfavorable immune responses and serve as an intriguing platform toward nucleic acids-based regenerative medicine.

On the hand, to translate this gene therapy strategy into clinical trial, the unintended consequences as a result of long-time expressing angiogenetic factors should be considered. For instance, the potential patients also suffering from the angiogenic diseases (e.g. tumors, wet age-related macular degeneration) or susceptible to VEGF therapy (e.g. diabetes, pregnancy) should be excluded from the clinical candidates. Moreover, it is important to investigate the possibilities of regenerating blood vessels distant from the injected peripheral arterial diseases, with the aim of avoiding angiogenesis of the fundamental organs (e.g. hearts, brains, livers, lungs, kidneys or spleens). Despite the aforementioned concerns, the proposed gene therapy strategy using pDNA is speculated to be relatively safe in view of the expressing period ranging from 2 to 3 weeks for our proposed pDNA vector. In addition, safety profiles could be further improved by carefully adjusting dosage and pDNA sequence (e.g. promotor) for minimizing the occurrence of the unfavorable over-angiogenesis.

## Conclusions

To this respect, the present study proposes the employment of intriguing virus-like toroidal pDNA condensates, which allow efficient in vivo gene expression at the gene therapy injection location. In evaluating the angiogenic VEGF pDNA payload, angiogenesis was verified to have been successfully accomplished, indicating successful recovery from peripheral arterial ischemia. Note that the current therapies deemed pertinent for peripheral arterial diseases involve bypass grafting, angioplasty, and atherectomy, which have made the treatment for critical late-stage ischemia exceedingly challenging. Therefore, our proposed angiogenetic gene therapy might stimulate neo-vasculature from the existing active arteries as an alternative to rescuing the impaired vasculature; this is consequently envisioned to provide a powerful tool in the clinical treatment of occlusive cardiovascular diseases with high-grade severity, e.g. myocardial ischemia and cerebrovascular diseases. Furthermore, our proposed PEGylated pDNA condensates was verified to substantially reduce pro-inflammation responses in relative to the naked pDNA, and this result is consistent with the crucial role of extracellular DNA in exacerbating arthritis. Pertaining to the recently approved vasculature regeneration gene therapy using high dose of pDNA (Collategene, AnGes), our proposed systems should not only enhance the therapeutic efficacies with much reduced dose but also improve the overall safety profiles.

### Electronic supplementary material

Below is the link to the electronic supplementary material.

Supplementary Material 1

## Data Availability

All data generated or analyzed during this study are included in this published article.
