# Supplementary material for: Gene therapy by virus-like self-spooling toroidal DNA condensates for revascularization of hindlimb ischemia
Source: J Nanobiotechnology. 2024 Jul 15;22:413. doi: 10.1186/s12951-024-02620-3 (PMC11247739; doi:10.1186/s12951-024-02620-3)
Supplement: Supplementary file 1 — Supplementary Material 1 [file 12951_2024_2620_MOESM1_ESM.pdf]

Gene Therapy by Virus-like Self-Spooling Toroidal DNA Condensates for Revascularization of Hindlimb Ischemia

Yue Wang[a,b,c,#], Jun Liu[d,e,#], Changgui Tong[f], Lei Li[f], Hongyang Cui[e], Liuwei Zhang[e], Ming Zhang[a,b], Shijia Zhang[g,h,i,j], Kehui Zhou [g,h,i,j], Xiabin Lan[g,h,i,j*], Qixian Chen[c,e,*], Yan Zhao[a,b,c,*]

[a]Department of Gastric Surgery, Cancer Hospital of China Medical University, No. 44 Xiaoheyan Road, Dadong District, Shenyang City, Liaoning 110042, China

[b]Department of Gastric Surgery, Cancer Hospital of Dalian University of Technology, No. 44 Xiaoheyan Road, Dadong District, Shenyang City, Liaoning 110042, China

[c]Provincial Key Laboratory of Interdisciplinary Medical Engineering for Gastrointestinal Carcinoma, Liaoning Cancer Hospital & Institute, No. 44 Xiaoheyan Road, Dadong District, Shenyang City, Liaoning 110042, China

[d]Department of Materials Science and Engineering, Tsuinghua University, Beijing City 100084, China

[e]Innovation Center of Yangtze River Delta, Zhejiang University, Jiaxing, Zhejiang 314100, China

[f]Department of Vascular Surgery, The Second Affiliated Hospital of Dalian Medical University, Dalian, Liaoning 116023, China

[g]Department of Thyroid Surgery, Zhejiang Cancer Hospital, Hangzhou, Zhejiang 310022, China

[h]Hangzhou Institute of Medicine (HIM), Chinese Academy of Sciences, Hangzhou, Zhejiang 310022, China

[i]Key Laboratory of Head & Neck Cancer Translational Research of Zhejiang Province, Hangzhou, Zhejiang 310022, China

[j]Postgraduate training base Alliance of Wenzhou Medical University (Zhejiang Cancer Hospital), Hangzhou, Zhejiang, 310022, China

*All correspondence should be addressed to Xiabin Lan (lanxb@zjcc.org.cn), Qixian Chen (plasmid@zju.edu.cn) and Yan Zhao (E-mail: zhaoyan@cancerhosp-ln-cmu.com).

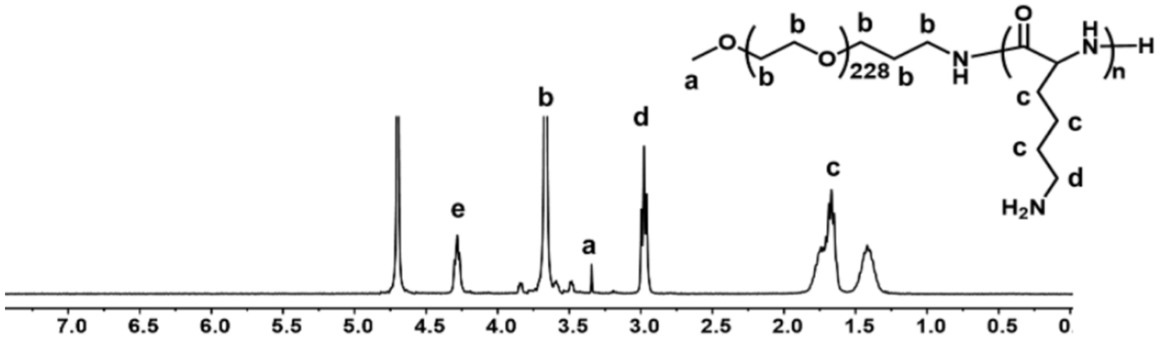

Figure S1 ^{1}H NMR spectrum of block copolymer of PEG-PLys in D_2O.

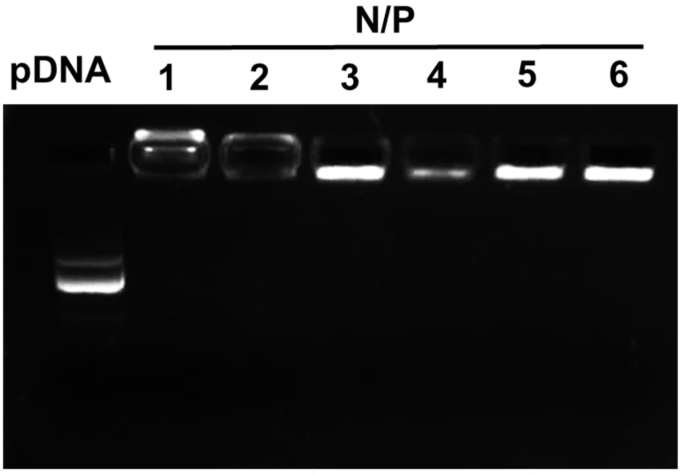

Figure S2. Gel electrophoresis for pDNA condensates at varied N/P ratios.

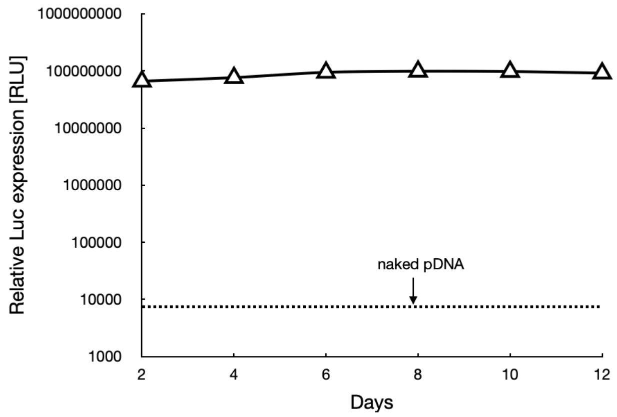

Figure S3. Real-time gene expression efficiencies of Luc by toroidal pLuc condensates by Kronos measurement.

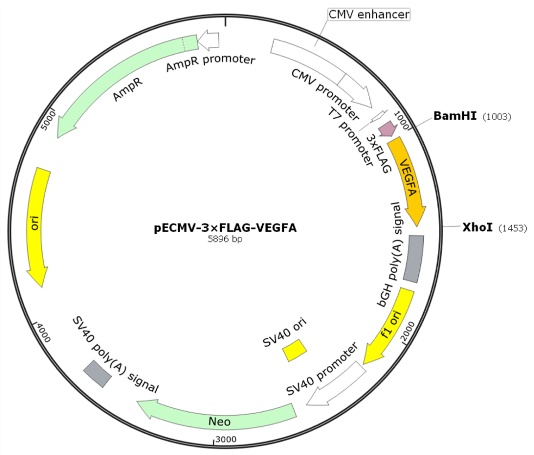

Figure S4. Genomic sequence of pVEGF.

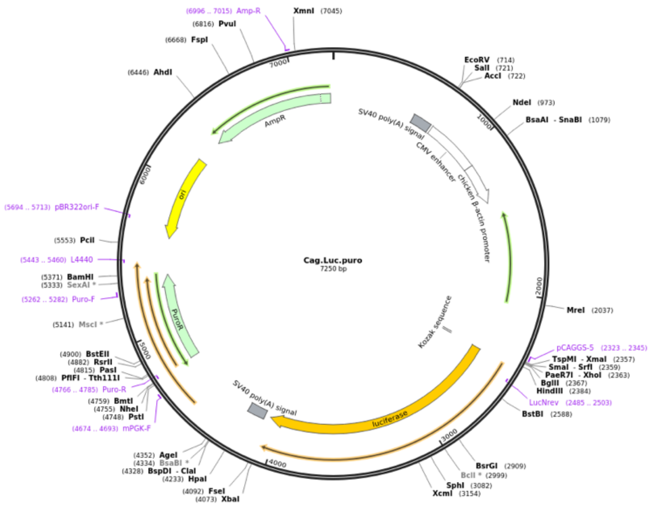

Figure S5. Genomic sequence of pLUC.
